# First draft genome and transcriptome of *Cercosporidium personatum*, causal agent of late leaf spot disease of peanut

**DOI:** 10.1186/s13104-023-06331-0

**Published:** 2023-04-21

**Authors:** Renee S. Arias, John T. Dobbs, Jane E. Stewart, Emily G. Cantonwine, Valerie A. Orner, Victor S. Sobolev, Marshall C. Lamb, Alicia N. Massa

**Affiliations:** 1 United States Department of Agriculture (USDA), Agricultural Research Service (ARS), National Peanut Research Laboratory (NPRL), 1011 Forrester dr. S.E., Dawson, GA USA; 2 Department of Agricultural Biology, Colorado State University, Fort Collins, CO USA; 3 Department of Biology, Valdosta State University, Valdosta, GA USA

**Keywords:** Late leaf spot, Genome, RNA polymerase, Ribosomal RNA, Peanut, *Cercosporidium*, *Nothopassalora*

## Abstract

**Objective:**

Two main fungal leaf spot diseases occur in peanut, namely early leaf spot (ELS) and late leaf spot (LLS), these cause a yearly average of $44 million losses. Limited genetic information, 3534 bp of sequencing, exists about the causal agent of LLS, *Cercosporidium personatum* (syn. *Nothopassalora personata*, syn. *Phaeoisariopsis personata*). The extremely slow growth of this fungus, approximately 1 cm colony in 6 months, and challenges in nucleic acid extractions have hindered research on LLS. Our goal in this work is to provide a reference genome for research on this pathogen.

**Results:**

Whole genome and transcriptome sequencing of the LLS fungus were obtained. A total of 233,542,110 reads of the genome were de novo assembled resulting in 1061 scaffolds, and estimated genome size 27,597,787 bp. RNA sequencing resulted in 11,848,198 reads that were de novo assembled into 13,343 contigs. Genome annotation resulted in 10,703 putative genes. BUSCO analysis of the genome and annotation resulted in 91.1% and 89.5% completeness, respectively. Phylogenetic dendrograms for 5442 bp and 4401 bp of RNA Polymerase II largest and second largest subunits, and for 5474 bp of the ribosomal RNA cistron of *C. personatum* are presented in relation to closely related fungi.

## Introduction

**Fig. 1:**
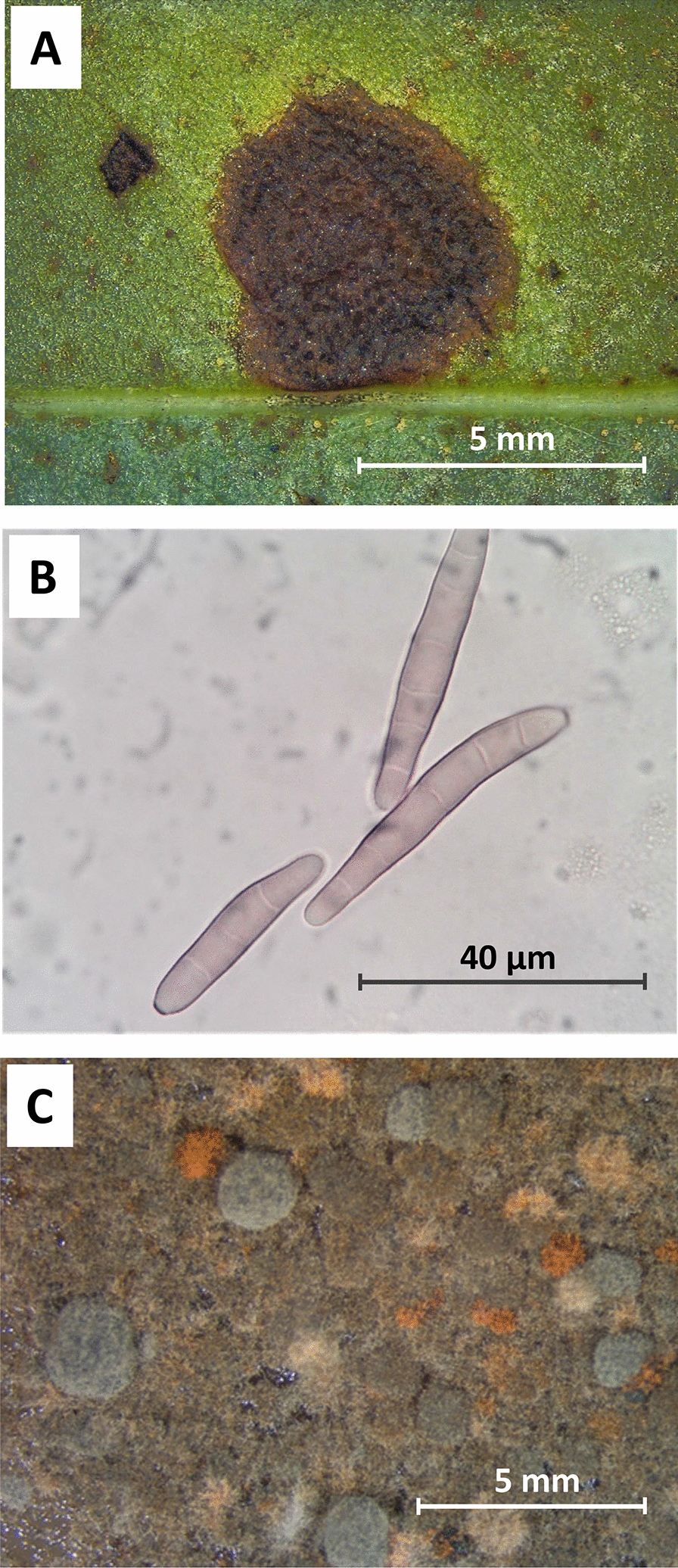
Peanut late leaf spot caused by *Cercosporidium personatum*. **A**: sporulating lesion in the abaxial surface of the leaf; **B**: conidia of *C. personatum* at 1000X; **C**: culture of *C. personatum* on PDA medium

Late leaf spot (LLS) disease (Fig. 1A) caused by the Ascomycete *Cercosporidium personatum* (Berk. & M.A. Curtis) Deighton (syn. *Nothopassalora personata*, syn. *Phaeoisariopsis personata*, syn. *Mycosphaerella personata*) is one of the two main leaf spot diseases reported for peanut plants (*Arachis hypogaea* L.). The other is early leaf spot (ELS), caused by *Cercospora arachidicola* S. Hori (syn. *Passalora arachidicola*, syn. *Mycosphaerella arachidis*). In the present article, we refer to the LLS agent as *C. personatum*, name widely recognized by the peanut community. Genetic information about *C. personatum* (listed as *N. personata*) is almost negligible in world databases; NCBI-GenBank lists only five gene fragments, a total of 3534 bp as follows: 1122 bp of DNA dependent RNA Polymerase II second largest subunit, 1069 bp of rRNA subunits/ITS regions, 451 bp of calmodulin, 354 bp of histone-H3, 321 bp of elongation factor 1-alpha and 217 bp of the actin gene.

ELS and LLS cause severe losses in peanut costing Georgia farmers $ 44 million a year [1] as consequence of reduced yield and expensive multiple fungicide applications [2–4]. Although the search for LLS resistance in peanut is a persistent effort [2, 5, 6], little is known about the population genetics of the pathogen, which is necessary to attain durable host resistance [7]. The availability of a *C. personatum* genome will enable such studies. In addition, limited morphological distinction within Ascomycetes has shown RNA Polymerase II as a more useful taxonomic tool [8]. A draft genome will also allow for a more robust phylogenetic analysis of this species, and perhaps settle its systematic placement, which has changed in recent years. Here we present the first reference genome and transcriptome of *C. personatum* to support studies in phylogenetics, genetic diversity, pathogenicity, and metabolites of this peanut pathogen.

## Main text

### Materials and methods

#### Isolation and growth

*C. personatum* CPDP-13A was obtained from a single spore isolated from Georgia-06G peanut leaves with late leaf spot disease, Fig. 1A. The abaxial side of a peanut leaf infected with late leaf spot disease was slid across the surface of a water agar plate to deposit spores, Fig. 1B. Single germinated spores matching the shape and size of *C. personatum* conidia were transferred to plates containing potato dextrose agar medium (PDA) (BD Difco 254920 + 15 g/L agar) as described previously for *C. arachidicola* [9]. After 6 months of growth under continuous light, a fragment of one colony, isolate CPDP-13A, was used for a DNA extraction using DNeasy Plant Mini Kit (Qiagen, MD), followed by PCR amplification of the internal transcribed spacer (ITS) region using primers ITS5 and ITS4 [10], and sequenced to confirm species identification. The remaining colony was shredded as reported previously for *C. arachidicola* [11], approximately 1 cm^2^ of stroma was placed in a sterile test tube with 2 mL sterile distilled water, and ground using a LabGEN 125 tissue homogenizer (Cole Parmer, Vernon Hills, IL, USA). Aliquots of 200 µL homogenate were spread on PDA plates, allowed to dry, sealed with parafilm and incubated at 26 °C with continuous light for 2 weeks.

#### DNA extraction for sequencing

Spores, conidiophores, and young hyphal fragments were scrapped from the surface of the PDA plates, collected into a 50 mL polypropylene centrifuge tube and ground in a Kleco tissue pulverizer (Garcia Machine, Visalia, CA, USA) as previously described [11]. Genomic DNA was extracted from the material using phenol/chloroform/isoamyl alcohol followed by isopropanol precipitation [12]. The genomic DNA was sequenced at the Broad Institute of MIT and Harvard (Cambridge, MA), as pair end (PE) 100 bp using Illumina HiSeq 2500 whole-genome shotgun approach, and reads were de novo assembled using ALLPATHS [13].

#### RNA extraction

A colony of CPDP-13A was homogenized with a LabGEN 125 as indicated above, and 200 µL of the homogenate were plated on PDA or PDA supplemented with 200 µL sterile V8 per plate (PDA + V8). Two media were used seeking to capture higher diversity of RNA molecules. The surfaces of the media were allowed to dry in a laminar flow until no free water was observed, then the plates were sealed with parafilm and incubated at 25 °C in the dark for 2 weeks. Pieces of approximately 1 cm^2^ of colonies from each media were placed in 2 mL tubes and ground twice for 30 s in an Omni Bead Ruptor 24 (Omni International, Kennesaw, GA, USA) with one min incubation on ice between cycles, and total RNA was extracted using the RNeasy Plant Mini Kit in a QIAcube robot (Qiagen, Redwood City, CA, USA). After quantitation, both RNA samples were combined in equal amounts for RNA sequencing and processed at LC Sciences (Houston, TX, USA) as 100 bp PE using Illumina HiSeq 2500. Reads were trimmed of potential adapters and assembled using CLC Genomics Workbench 20.0.4 (Qiagen, Aarhus, Denmark).

#### Cercosporidium genome annotation

The *C. personatum* genome was annotated using the MAKER v2.31.8 annotation pipeline [14] with RepeatMasker v4.0.8 [15]. A genome-specific repeat library was constructed using RepeatModeler v1.0.11 [16] to mask interspersed repeats and low complexity DNA sequences. Three gene predictors were used in the pipeline: GeneMark-ES [17], SNAP [18], and AUGUSTUS [19]. *Neurospora crassa* was used as a species model for AUGUSTUS. The *C. personatum* transcriptome was used along with the ab initio gene predictors as evidence for the annotation. To identify tRNA genes, tRNAscan-SE v1.3.1 [20] was used with default settings. Predicted transcripts (≥ 150 bp) and proteins (≥ 50 amino acids) were analyzed using five databases for analysis of putative genes: InterProScan for protein family domains [21], SignalP for signal peptides [22], EffectorP for apoplastic and cytoplasmic effectors [23], antiSMASH 5.0 for putative secondary metabolites [24], dbCAN2 for putative carbohydrate-active enzymes (CAZymes) [25], and PHI-base for putative virulence-associated proteins [26]. antiSMASH, and dbCAN2 online servers were used to analyze the predicted genes at default settings. Only proteins with dbCAN2 hits in at least two of the three databases (HMMER, DIAMOND and e-CAMI) were considered. SignalP, EffectorP, InterProScan, & PHI-base were assessed locally. Selection of putative proteins from PHI-base was based on a ≥ 60% percent identity. The quality assessment and completeness of the genome assembly and annotation were assessed locally using QUAST [27] for genome statistics and the BUSCO conserved genes for the order Capnodiales data set for completeness of conserved genes [28].

## Results

### Cercosporidium genome and transcriptome assembly

The single-spore *C. personatum* isolate CPDP-13A was stored in the ARS Culture Collection, Northern Regional Research Laboratory, Peoria, Illinois, with Accession number NRRL 64463. A total of 233,542,110 Illumina HiSeq reads (100 base PE), Table 1, DataSet_1 [29], obtained from *C. personatum* NRRL 64463 were originally assembled at the Broad Institute into 3077 contigs, 2347 scaffolds (NCBI accession LIHA00000000). After further manual editing, the number of scaffolds was reduced to 1061, with N50: 47,859, GC content 53%, largest scaffold 258,482 bp, Table 1, Data_file_1 [30]. BUSCO of conserved gene set within phylum Ascomycota and within order Capnodiales for the genome assembly showed a 93.2 and 91.1% completeness, respectively; whereas the completeness of the genome annotation for those databases was 90.8 and 89.5%, respectively. The estimated genome size was 27,598,742 bp with an average coverage of 625 X. The transcriptome sequencing resulted in 11,848,198 reads (100 base PE) that assembled to 13,343 contigs (≥ than 300 bases), Table 1, DataSet_2 [31], Data_file_2 [32].

**Table 1:** Summary statistics and files for the genome and transcriptome of *Cercosporidium personatum*

Label	Name of data file/data set	File type	Data repository and identifier (DOI or accession number)
Genome description			
Data set 1	SRR22033838	.SRA	^a^NCBI: sequence read archive. https://www.ncbi.nlm.nih.gov/sra/SRR22033838 [29]
Data file 1	CP_Genome	.FASTA	^b^HD: https://doi.org/10.7910/DVN/NZDNCY [30]
Data file 6	Predicted_gene_min150	.FASTA	HD: https://doi.org/10.7910/DVN/LUTTV5 [36]
Data file 7	Putative_proteins_min50	.FASTA	HD: https://doi.org/10.7910/DVN/PNEDNI [37]
Data file 8	InterProScan	.TSV	HD: https://doi.org/10.7910/DVN/E9EOAU [38]
Data file 9	Genome_EffectorP	.TXT	HD: https://doi.org/10.7910/DVN/M6QDZH [39]
Data file 10	Genome_dbCAN2	.XLSX	HD: https://doi.org/10.7910/DVN/UUVFIY [40]
Data file 11	Genome_antiSMASH	.XLSX	HD: https://doi.org/10.7910/DVN/RMBQ0E [41]
Data file 12	Genome_PHIBLAST	.XLSX	HD: https://doi.org/10.7910/DVN/D15NQC [42]
Genomic data for phylogenetics			
Data file 3	rRNAcistron_CP&others_5474bp	.FASTA	HD: https://doi.org/10.7910/DVN/V9MU3N [33]
Data file 4	RPB1_CP&others_5442bp	.FASTA	HD: https://doi.org/10.7910/DVN/4KHA0I [34]
Data file 5	RPB2_CP&others_4401bp	.FASTA	HD: https://doi.org/10.7910/DVN/QBNTFZ [35]
Transcriptome description			
Data set 2	SRR 22077678	.SRA	NCBI: sequence read archive. https://www.ncbi.nlm.nih.gov/sra/SRR22077678 [31]
Data file 2	Contigs_300b_13343	.FASTA	HD: https://doi.org/10.7910/DVN/82DHQI [32]
Data file 13	Transcriptome_EffectorP	.TXT	HD: https://doi.org/10.7910/DVN/UUH9R7 [43]
Data file 14	Transcriptome_dpCAN2	.XLSX	HD: https://doi.org/10.7910/DVN/HS5YEO [44]
Data file 15	Transcriptome_PHIBLAST	.XLSX	HD: https://doi.org/10.7910/DVN/X4BXGD [45]

^a^NCBI: National Center for Biotechnology Information

^b^HD: Harvard Dataverse

### Relevant sequences for phylogenetics of *C. personatum*

**Fig. 2:**
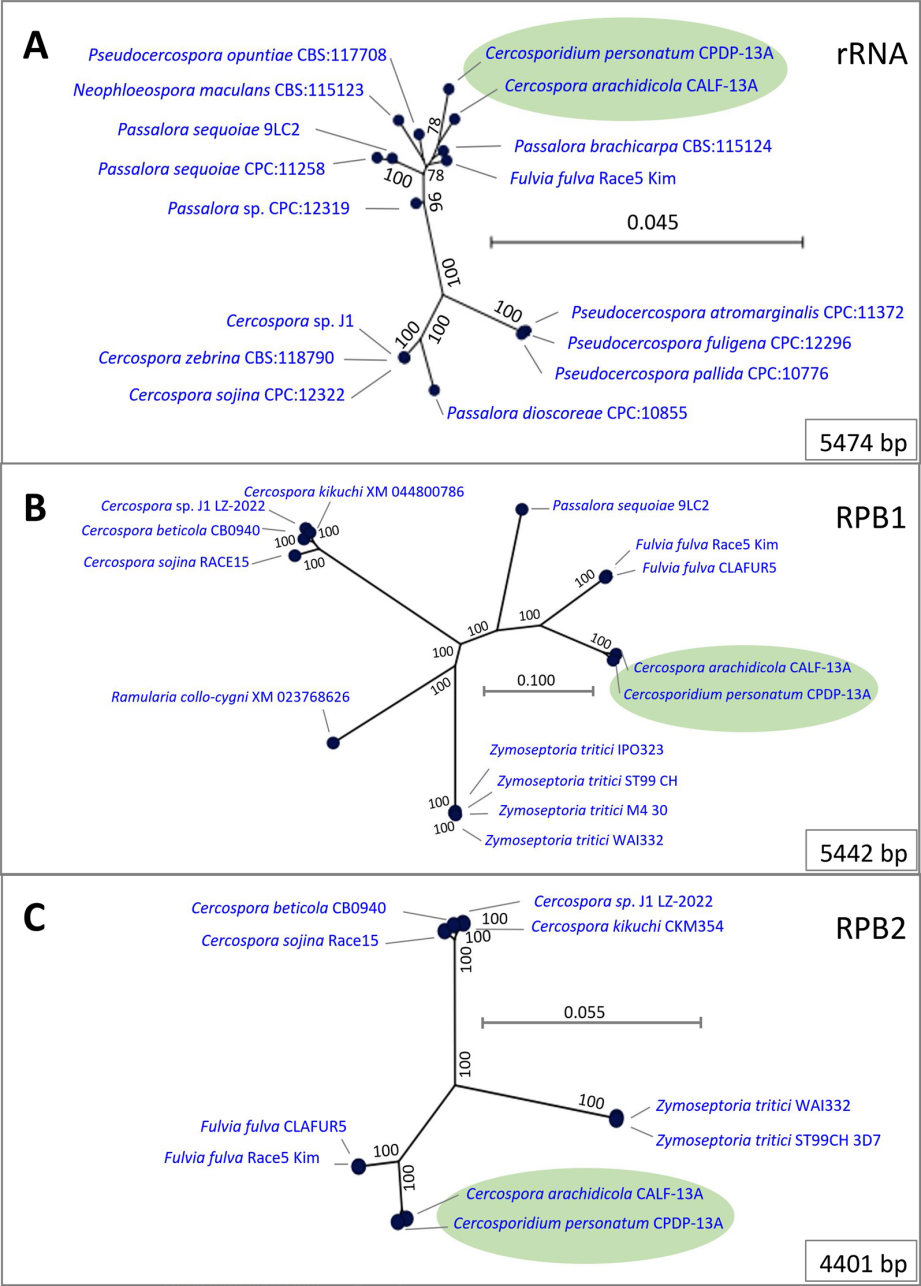
Phylogenetic dendrograms of *Cercosporidium personatum* in relation to closely related fungi. **A**: representation based on 5474 bp of the ribosomal RNA cistron; **B**: representation based on 5442 bp of the DNA dependent RNA Polymerase II (RNA Pol II) largest subunit; **C**: representation based on 4401 bp of RNA Pol II second largest subunit. Numbers at the nodes show the support of the branching using 100 replicate bootstrapping. Scale bars = nucleotide (nt) substitutions per 100 nt

Sequences of three loci commonly used in phylogenetics are reported here for NRRL 64463 in relation to closely related species. One is 5474 bp of the ribosomal RNA (rRNA) cistron that consists of most of the 18S, 5.8S, ITS1, ITS2 and 28S rRNA (Table 1, Data_file_3 [33], Fig. 2A. Another sequence is 5442 bp of the largest subunit of the DNA dependent RNA Polymerase II (RPB1) (Table 1, Data_file_4 [34], Fig. 2B). And the third one is a 4401 bp of the second largest subunit of RNA Pol_II (RPB2) (Table 1, Data_file_5 [35], Fig. 2C). The dendrograms of these three sequences, placed the agent of peanut LLS (*C. personatum*) phylogenetically close to the agent that causes peanut ELS (*C. arachidicola*), Fig. 2. In some cases, the genetic distances between these two organisms were similar to the distances between isolates of the same species for other Ascomycetes represented in the dendrograms, Fig. 2BC.

### *Cercosporidium personatum* genome annotation

We predicted from the genome a total of 10,703 putative genes (predicted transcripts ≥ 150 bases), and 10,694 putative proteins (≥ 50 amino acids), Table 1, Data_file 6 [36], Data_file 7 [37] in the 27.59 Mb genome of *C. personatum*. A total of 10,288 of the predicted genes had described protein family domains, Table 1, Data_file 8 [38]. A total of 753 (Table 1, Data_file_9 [39]) signal peptides were identified as part of the putative secretome. These were further described as 151 apoplastic and 127 cytoplasmic effectors, Table 1, Data_file_9 [39]. The putative CAZymes in decreasing order of abundance, were composed of glycoside hydrolases (GH; involved in hydrolysis and/or rearrangement of glycosidic bonds) (197 genes), glycosyl transferases (GT; involved in the formation of glycosidic bonds) (97 genes), auxiliary activities (AAs; are redox enzymes that act in conjunction with CAZymes) (61 genes), carbohydrate esterases (CE; involved in hydrolysis of carbohydrate esters) (20 genes), carbohydrate-binding modules (CBMs; involved in adhesion to carbohydrates) (15 genes), and polysaccharide lyases (PL; involved in non-hydrolytic cleavage of glycosidic bonds) (8 genes), Table 1, Data_file_10 [40]. Gene clusters involved in the production of dothistromin, and melanin were identified among the putative secondary metabolite gene clusters, Table 1, Data_file_11 [41]. Non-ribosomal peptide synthetase (NRPS) (9 genes) and NRPS-like (9 genes) clusters were the most represented secondary metabolite gene clusters followed by type I polyketide synthases (T1PKS) (4 genes) and terpenes (3 genes). Several other secondary metabolite gene clusters were identified including, fungal ribosomally synthesized and post-translationally modified peptide products (RiPPs), non-alpha poly-amino acids (NAPAA), siderophores, and T3 polyketide synthase clusters (1 gene each), Table 1, Data_file_11 [41]. A total of 876 putative virulence related proteins, were identified including virulence-related proteins of other members of Mycosphaerellaceae including *Fulvia fulva* [AOX1 (PHI: 199), Avr4 (PHI: 18), Avr5 (PHI: 5538)] and *Dothistroma septosporum* [DsAvr4 (PHI: 5477)], Table 1, Data_file_12 [42]. Further investigation of the transcriptome identified 189 signal peptides which were described as 25 apoplastic and 54 cytoplasmic effectors, Table 1, Data_file_13 [43]. Furthermore, six CAZymes including two GT and four GH were identified from the transcriptome, Table 1, Data_file_14 [44]. When investigating putative virulence associated proteins, 2286 proteins were described, with protein kinases and transcription factors identified as having the highest number of copies, 12 and 11 gene copies respectively, Table 1, Data_file_15 [45].

A 71% of transcriptome reads mapped to the assembled genome, 92% of these mapped as paired, using the parameters 50% read length, 80% identity, and gap/insertion cost = 3. In addition, 92% of the de novo assembled transcripts mapped to the genome.

## Discussion

The availability of the genome for the causal agent of LLS in peanuts will provide an important tool for plant pathologists and peanut breeders, as it can be used to develop more tools for assessing the level of genetic diversity of this pathogen in peanut growing areas. The data presented, are an initial step for studying other aspects of this pathogen. For example, variable color forms are observed for colonies that originated from single-spore cultures of *C. personatum*, Fig. 1C, [46, 47]; but so far, the lack of molecular tools has prevented research in this area. When using only 872 bp of RPB1, Schoch et al. found a 0.8 probability of correct identification (PCI) of Ascomycota, *Pezizomycotina*, and reported that RPB1 had a high level of discrimination in all fungal groups [10]; here we provided 5442 bp of RPB1. Likewise, a 559 bp region of the rRNA cistron that includes ITS1, ITS2 and 5.8S gene, had a PCI of 0.71, and was proposed as a DNA barcode for classification of fungi [10], here we provided 5474 bp of the rRNA cistron that includes the ITS region in addition to the 28S and 18S rRNA subunits. Since the genome of the ELS agent has already been sequenced [11], and phylogenetic analysis of the LLS agent placed both species in close proximity, Fig. 2, this report may contribute to a more stable taxonomy for these organisms.

## Limitations

*C. personatum* grows extremely slowly, forming an approximate 1 cm^2^ colony in 6 months. Nucleic acid extractions from its culture result in low yields. Despite being isolated from a single spore, cultures of *C. personatum* often present various colony colorations. Therefore, much research needs to be done to understand the biology of this plant pathogen.

## Data Availability

The raw data of genome and transcriptome described in the present note can be freely and openly accessed at the NCBI SRA Database, accessions SRR 22033838 and SRR 22077678, Table 1, Data sets 1 [29] and 2 [31], respectively. Please see Table 1 for details and links to data files 1 through 15 [30, 32–45].

## Abbreviations

b: Base
bp: Base pair
BUSCO: Benchmarking Universal Single-Copy Orthologs
ELS: Early leaf spot
ITS: Internal transcribed spacer
LLS: Late leaf spot
PCI: Probability of correct identification
PDA: Potato dextrose agar
PE: Paired ends
RNA: Ribonucleic acid
